# WD repeat domain 82 (Wdr82) facilitates mouse iPSCs generation by interfering mitochondrial oxidative phosphorylation and glycolysis

**DOI:** 10.1007/s00018-023-04871-z

**Published:** 2023-07-20

**Authors:** Guina Cui, Jingxuan Zhou, Jiatong Sun, Xiaochen Kou, Zhongqu Su, Yiliang Xu, Tingjun Liu, Lili Sun, Wenhui Li, Xuanning Wu, Qingqing Wei, Shaorong Gao, Kerong Shi

**Affiliations:** 1 Key Laboratory of Animal Bioengineering and Disease Prevention of Shandong Province, College of Animal Science and Technology, Shandong Agricultural University, No. 61 Daizong Street, Taian, 271018 China; 2 Clinical and Translational Research Center of Shanghai First Maternity and Infant Hospital, Frontier Science Center for Stem Cell Research, School of Life Sciences and Technology, Tongji University, Shanghai, 200092 China

**Keywords:** Reprogramming, Yamanaka factors, iPSC generation, Cell fate transition, Metabolic switching, Oxidative phosphorylation (OXPHOS)

## Abstract

**Background:**

Abundantly expressed factors in the oocyte cytoplasm can remarkably reprogram terminally differentiated germ cells or somatic cells into totipotent state within a short time. However, the mechanism of the different factors underlying the reprogramming process remains uncertain.

**Methods:**

On the basis of Yamanaka factors OSKM induction method, MEF cells were induced and reprogrammed into iPSCs under conditions of the oocyte-derived factor Wdr82 overexpression and/or knockdown, so as to assess the reprogramming efficiency. Meanwhile, the cellular metabolism was monitored and evaluated during the reprogramming process. The plurpotency of the generated iPSCs was confirmed via pluripotent gene expression detection, embryoid body differentiation and chimeric mouse experiment.

**Results:**

Here, we show that the oocyte-derived factor Wdr82 promotes the efficiency of MEF reprogramming into iPSCs to a greater degree than the Yamanaka factors OSKM. The Wdr82-expressing iPSC line showed pluripotency to differentiate and transmit genetic material to chimeric offsprings. In contrast, the knocking down of Wdr82 can significantly reduce the efficiency of somatic cell reprogramming. We further demonstrate that the significant suppression of oxidative phosphorylation in mitochondria underlies the molecular mechanism by which *Wdr82* promotes the efficiency of somatic cell reprogramming. Our study suggests a link between mitochondrial energy metabolism remodeling and cell fate transition or stem cell function maintenance, which might shed light on the embryonic development and stem cell biology.

**Supplementary Information:**

The online version contains supplementary material available at 10.1007/s00018-023-04871-z.

## Introduction

The mammalian oocyte cytoplasm includes abundant powerful reprogramming factors, which can remarkably reprogram terminally differentiated somatic cells into totipotent state within a short time. This reprogramming is the basic principle underlying somatic cell nuclear transfer (SCNT) and/or somatic cell cloning. Therefore, it has been previously speculated that oocyte-derived transcripts or proteins promote somatic cell reprogramming [35, 37]. It has also been suggested that Yamanaka factors (*Oct4*, *Sox2*, *Klf4*, *c-Myc*, collectively called OSKM factors) increase the speed and efficiency of the reprogramming process [33]. Induced pluripotent stem cells (iPSCs) have garnered considerable attention for use in patient-specific stem cell production and/or individualized treatment in mammalian animals, including scientists interested in their benefits for technical simplification and lack of ethics concerns. However, the current iPSC generation technology is still limited by bottlenecks, such as low induction efficiency, poor safety profile and long experimental period when compared with somatic cell nuclear transfer (SCNT) [17, 24]. To address the shortcomings of iPSC generation, the molecular mechanisms of SCNT have again attracted the interest of scientific researchers because of the similarity of cell fate reprogramming mediated by SCNT and iPS induction.

In fact, increasing evidence is revealing the molecular mechanisms underlying mouse embryonic fibroblast reprogramming, which is roughly categorized into initiation, maturation and stability stages. In the initiation stage of reprogramming, a wave of transcriptional and epigenetic modifications lead to changes in cell proliferation, metabolism and/or extracellular matrix remodeling. The acceleration of the cell cycle is conducive to reprogramming [14], for example, silencing of the tumor suppressor factor *p53* accelerated reprogramming [18] due to its inhibitory effects on cell cycle progression. *Zscan4* has been shown to prolong telomere life [23] and to act in conjunction with Yamanaka factors to inhibit the DNA damage response (DDR), stabilize genomic DNA and downregulate *p53*, thereby improving the efficiency of iPSC generation [16]. Regarding cellular metabolism reprogramming, *Tcl1* inhibits the mitochondrial localization of polynucleotide phosphorylase (PnPase), suppressing mitochondrial biogenesis and oxidative phosphorylation and promoting energy metabolism remodeling, thereby promoting reprogramming [19]. Glis1 enables reprogramming of senescent cells into pluripotent cells by binding to chromatin at glycolytic genes, thereby enhancing acetylation (H3K27Ac) and lactylation (H3K18la) at pluripotency gene loci and facilitating cellular programming [26], indicating the link between somatic cell reprogrramming and cell fate determination [27, 28]. For extracellular matrix remodeling, *Obox1*, that is, oocyte-specific homeobox 1, facilitates cell reprogramming by promoting the mesenchymal-to-epithelial transition (MET) process and attenuating cell hyperproliferation [37]. *Wdr5*, *Brca1* and *Bard1* work together in response to DNA damage, and their absence affects the MET in the early stages of reprogramming [31].

The mature and stable reprogramming stage is based mainly on epigenetic modifications and pluripotency network maintenances. *Dppa2* and *Dppa4*, key components of the chromatin-remodeling network, control pluripotency transformation by forming heterodimers, acting in conjunction with Yamanaka factors and remodeling chromatin configuration, thereby enhancing reprogramming [15]. The DNA methylation hydroxylase *Tet1* cooperates with *Oct4* in reprogramming and thus produce high-quality iPSCs [5]. Knockout of histone deacetylase 2 (HDAC2) promotes pluripotency factor binding to TET1, thereby reducing the DNA demethylation rate. Studies have shown that HDAC2-TET1 is a switch that regulates the maturation of pre-iPSCs into iPSCs [36]. Changes in epigenetic modifications affect the reprogramming stage. *Zscan4f*, a member of the ZSCAN protein family, recruits TET2. The Zscan4f-TET2 interaction promotes DNA demethylation and regulates the expression of downstream target genes associated with cellular metabolism and proteasome function, ultimately promoting iPSC generation [6].

In terms of epigenetic modification and pluripotency gene expression, WD repeat domain 82 (Wdr82) attracted our attention. First, Wdr82 has been identified as an oocyte-derived protein via mass spectrometry and RNA sequencing and is abundantly expressed in MII oocytes [12, 35], and the highly expressed oocyte cytokines had been reported to promote reprogramming. Second, the absence of Wdr82 reduces the expression level of pluripotent gene *Oct4*, hindering embryonic growth and development, and even leading to embryonic death [3]. On the other hand, the expression level of *Wdr82* is significantly reduced during mESC differentiation, and the cell proliferation rate is significantly slowed after *Wdr82* knockdown, indicating that *Wdr82* plays an important role in mESC proliferation and differentiation in vitro [3, 42]. Wdr82 is a C-terminal domain-binding protein that recruits the Setd1A/B histone H3K4 methyltransferase complex and RNA polymerase II. The Wd82 component of the complex interacts not only with the RNA recognition motif of Setd1A but also the phosphorylated C-terminal domain of RNA polymerase II, thereby recruiting the Setd1A/B complex to the transcription starting sites of certain genes and catalyzing the histone H3K4 trimethylation of adjacent nucleosomes, activating their transcription [25, 38]. The loss of Wdr82 specificity resulted in a significant reduction of H3K4 trimethylation level of transcriptional genes, such as *Oct4* [3, 38]. Hence, whether the highly expressed oocyte factors can increase the reprogramming efficiency of mouse somatic cells and the mechanism of Wdr82 function in iPSC generation become the foci of our attention. Here, we show that the overexpression of *Wdr82,* as the basis of OSKM factor-mediated reprogramming, significantly promotes the generation of iPSCs. Further analysis indicates that the overexpression of Wdr82 affects the cellular metabolic pathway by inhibiting oxidative phosphorylation in mitochondria during the early stage of somatic cell reprogramming.

## Materials and methods

### Ethical statement

All animal experiments were carried out according to the Regulations for the Administration of Affairs Concerning Experimental Animals published by the Ministry of Science and Technology, China (2004). All animal maintenance and experimental procedures were performed according to the Tongji University Guide for the use of laboratory animals.

### Contact for reagent and resource sharing

Further information and requests for resources and reagents should be directed to and will be fulfilled by the corresponding author, Shaorong Gao (gaoshaorong@tongji.edu.cn).

### Animals

ICR pregnant mice were purchased from the Beijing Sibeifu Company. C57BL/6 mice, OSKMOR transgenic mice and *Oct4*−GFP mice were purchased or otherwise obtained from Beijing Weitong Lihua Company, Shanghai Slack Laboratory Animal Co., Ltd., and the Jackson Laboratory, respectively. All mice involved in the study were SPF (specific pathogen-free) animals.

### Plasmids

A pFuw-TetOn retroviral vector was used as the backbone to construct plasmids expressing *Oct4*, *Sox2*, *Klf4*, *c-Myc*, *Wdr82*, *Dpy30* or *Taf7*. The primer pairs used to prepare constructs are listed in Table S1. *Wdr82*-shRNA were cloned into a psiccoR-RFP retroviral vector. The shRNA sequences are listed in Table S2.

### Reprogramming to transition MEFs into iPSCs

One day before infection, OR-MEF/OG2-MEF cells were resuscitated and inoculated at 2.0 × 10^4^ cells/well into a 12-well plate. The cells were then transfected with HEK293 cell-produced viral fragments carrying fusions of *Oct4*, *Sox2*, *Klf4* and *c-Myc* genes (named OSKM factors) and/or *Wdr82*, with 5 μg/mL polybrene*.* After infection for 10–12 h, the culture medium was replaced to ESM containing Dox, and reprogramming induction was initiated, which was recorded as Day 0. Then, the culture medium was changed using replaced with fresh ESM medium supplemented Dox the next day, depending on the colony situation. Dox was removed until a large number of *Oct4*-GFP fluorescence-emitting clones were observed. Subsequent experiments, including flow cytometry and AP staining, and the establishment of the OSKM + *Wdr82* iPSC line realized by selecting monoclonal cells, were performed when a *Oct4*-GFP-positive clone culture remained stable for three days. During the reprogramming process, the colony number was recorded every day, and *Oct4*-GFP-positive clones were also observed and imaged with a fluorescence microscope (IX73, Olympus, Japan).

### RT-PCR

A PCR system was prepared on ice, and each sample was prepared as three biological replicates. Every PCR was performed using 2 × RealStar Fast SYBR qPCR Mix (HIGH ROX) (GenStar, A303-05) with primers and cDNA (5 ng). The reactions were run in triplicate on a fluorescence quantitative detection system (Bio–Rad) following the manufacturer’s instructions. Cycle threshold values were normalized to murine housekeeping gene *Gapdh* or *Hprt* mRNA expression. The primer pairs used for assessing the expression of exogenously added and/or endogenously expressed *Oct4*, *Sox2*, *Klf4*, *c-Myc* and other pluripotency genes are listed in Tables S3 and S4.

### Flow cytometry

After the reprogramming induction period was completed, the original medium was removed by aspiration, and the cells were washed 1–2 times with DPBS; 300 μL of trypsin was used for digestion; and the same amount of FM medium was added to stop the trypsin digestion; and then, the cells were centrifuged at 1000 rpm at room temperature for 5 min. Next, 1 mL of the cell sample resuspended in DPBS was obtained, and computer-based analysis and detection of *Oct4*-GFP-positive and *Oct4*-GFP-negative cells were identified according to the BD LSRFortessa flow cytometry-based computer operation manual.

### Alkaline phosphatase (AP) staining

After the completion of reprogramming induction, well-conditioned iPSC clones were selected for AP staining. The clones were washed 3 times with DPBS, and then 4% paraformaldehyde was added to fix the cells for 5 min or overnight at 4 °C. Then, the fixative was discarded, and the cells were washed 3 times with DPBS. A staining solution was then added to the cells in the dark, followed by washing with DPBS. The AP staining results were imaged, and the number of AP-positive clones was counted.

### Embryoid body differentiation experiment

The hanging drop method was used to perform a EB differentiation experiment. The confluent iPSCs were trypsinized into single cells, followed by the removal of feeder cells. Then, the cell density was determined and adjusted to 5 × 10^4^/mL in ESM (without LIF) after gentle pipetting. The cell suspension was evenly arranged on the lid of a 10-cm cell culture dish at a concentration of 20 μL cell suspension/drop; after 48 h of incubation, the formation of EB spheres in the hanging drop was observed. The EBs were collected and treated with 0.1% gelatin in a six-well plate, with the medium was changed every other day. Then, the cells were cultured in a 37 ℃ incubator for 3 days (the EB spheres were allowed to adhere to the plate walls and differentiate). At the end of the differentiation period, total RNA was extracted from the differentiated cells and reverse transcribed to evaluate germ layer marker gene expression. The primer pairs used for the assessment of the marker genes in the differentiated germ layers are listed in Table S5.

### Immunofluorescence staining

The iPSC line was cultivated on a cell slide in a 24-well plate until the clone reached the proper density and size had grown to a suitable size. Then, the ESM medium was aspirated, and the cells were washed with DPBS, fixed at room temperature for 2 h, permeabilized with 0.1% Triton X-100 solution for 15 min at room temperature, blocked with a 3% BSA solution for 1 h at room temperature, incubated with primary antibody and secondary antibody for 1 h, and incubated with DAPI nuclear-staining solution for 5–10 min. Then, antifluorescence quenching solution was dropped on each slide. Each cell slide was placed face down, onto a glass slide, and finally sealed with nail polish. The slides were scanned and imaged with a Leica TCS SPE confocal microscope.

The following primary antibodies were used: anti-SSEA1 (Abcam, ab16285), anti-NANOG (CST, #8822S), anti-SOX2 (Abcam, ab97959) and anti-H3K4me3 (Bioss, bsm-33110 M). The following secondary antibodies were used: goat antimouse IgG Alexa Fluor 594 (Life, A-11005) and donkey antirabbit IgG Alexa Fluor 594 (Life, A-21207).

### Karyotype analysis

The cultured iPS cell lines (OSKM + Wdr82 and/or OSKM) are removed the original culture medium and add culture medium containing 0.25 g/mL colchicine (sigma, C-9754), incubating for 4–8 h until most clones show grape-like shape, being arrested in the metaphase. Then the colonies are digested into single cells by using trypsin at 37 ℃, followed by the trypsin neutralization and centrifuge at 1000 rpm for 5 min. Discard the supernatant, and resuspend the cells in 1 mL of preheated hypotonic solution (0.4 M sodium citrate and 0.4 M KCl in a 1:1 ratio) and then incubate in a 37 ℃ water bath for 5 min. Then the cells are prefixed and fixed by adding seven drops and 4 mL of fixation solution (methanol: glacial acetic acid in a 3:1 ratio), respectively, for 40 min at room temperature. After the second fixation, centrifuge at 1000 rpm for 5 min, discard the supernatant, and add 50 μ L fixation solution, gently resuspend cell precipitation using a Babbitt tube. The cell suspension are dropped onto the precooled glass slides from a height of approximately 1.5 m. Place the glass slides in a 65 ℃ oven for more than 2 h, followed by Giemsa staining (dilute 10 times of Giemsa concentrate as the working solution) in a staining tank for 15 min in a 37 ℃ oven. Observe and find the division phase where chromosomes are evenly separated under a microscope. Capture and analyze the number of chromosomes in 20 metaphases for each iPS cell line (Fig. S2). In total, 10 OSKM + Wdr82 iPSC lines and 5 OSKM iPSC lines were assessed for normal karyotype percentage.

### Chimeric mouse experiment

Chimeric mice were produced with a white recipient mouse embryo with an ICR background. Chimeric embryos were obtained via the 8-cell embryo polymerization method and transplanted into a mouse uterus. The chimeric mice were born after 13.5 days in utero via cesarean section upon veterinary anesthesia using avertin (soamyl alcohol and tribromoethanol mixture in an equal proportion, followed by 50 times dilution, and inject at 0.15 times the body weight of the mouse). Because the donor OSKM-*Wdr82*-iPS cells carried a black C57 background, the adult chimeric mice presented with a black and white coat color (Fig. S3).

### RNA-seq

Three sets of biological replicate cell samples collected on reprogramming Day 3, Day 6 and/or Day 9, as well as the original somatic MEF cells, were subjected to RNA-seq. These samples were named as follows: Wdr82-1, Wdr82-2, and Wdr82-3 and Vector-1, Vector-2, and Vector-3 for the cell samples collected at reprogramming Day 3 from the OSKM + Wdr82 and OSKM groups; similarly, Wdr82-4, Wdr82-5, and Wdr82-6 and Vector-4, Vector-5,and Vector-6 were the names of the samples obtained on reprogramming Day 6, and Wdr82-7, Wdr82-8, and Wdr82-9 and Vector-7, Vector-8, and Vector-9 were the names of the samples obtained on Day 9. MEF-1, MEF-2, and MEF-3 were the negative control cells. Genes that were differentially upregulated or downregulated during the reprogramming process were identified based on a cutoff *P* value < 0.05 and |log2(fold-change)|≥ 1. Heatmaps were drawn using Heatmapper (http://www.heatmapper.ca/). KEGG and Gene Ontology Biological Process analyses were performed using the DAVID gene set enrichment tool. The primer pairs used for qRT‒PCR to validate of the identification of the differentially expressed genes associated with OXPHOS are listed in Table S6.

### MMP assay

Mitochondrial membrane potential (MMP) was assessed using the mitochondrial membrane potential assay dye JC-1 according to the manufacturer's protocol (Beyotime, C2006, Nanjing, China). A total of 5 × 10^5^ cells were washed twice with PBS and incubated with JC-1 dye in serum-free medium for 20 min at 37 °C. After washing twice, the cells were analyzed using a BD Accuri C6 flow cytometer or observed under a microscope (IX73, Olympus, Japan). In normal mitochondria, JC-1 aggregates in the mitochondrial matrix to form a polymer, which emits intense red fluorescence (Em = 590 nm), while in cells with lost membrane potential in unhealthy mitochondria, JC-1 is found only as monomers in the cytoplasm, emitting green fluorescence (Em = 529 nm).

### ROS detection

ROS generated in cells were detected using a DHE probe (Applygen, C1300-2, Beijing, China). Cells in the supernatant were removed and incubated at 37 °C for 30 min in DHE probe solution diluted with serum-free culture medium. The upper layer of the culture medium was discarded, and the cells were washed with DPBS, and then observed under a microscope (Em = 520 nm).

### ATP production quantitation

The ATP amount produced in cells was assessed according to the standard protocol (Beyotime, S0026, Nanjing, China). Cells were lysed in lysis buffer, and then the supernatant was taken for subsequent determination after centrifugation for 5 min at 4 ℃. The supernatant was incubated for 5 min in a proportional ATP detection working solution at room temperature, followed by equal mixing with the standard solution and measurement of the RLU or CPM value with a chemiluminescence meter. The ATP value in OSKM-*Wdr82* cells was obtained after normalization of the background and the subsequent OSKM control experiments**.**

### MitoTracker staining assay

Cells on coverslips in a petri dish were removed from the culture medium and then incubated for 20 min in a prewarmed staining solution containing a MitoTracker probe (Thermo Fisher Invitrogen, M22426). The staining solution was then replaced with fresh prewarmed medium, and cells were observed under a fluorescence microscope (Em = 520 nm).

### Statistical analyses

All data are reported as the means ± SEMs. The results were analyzed using Student’s *t* test. Differences were considered statistically significant when* P* < 0.05. At least three independent experiments were performed in replicates for statistical analyses. *P < 0.05, **P < 0.01 and ***P < 0.001.

## Results

### *Wdr82* increases somatic cell reprogramming efficiency

**Fig. 1:**
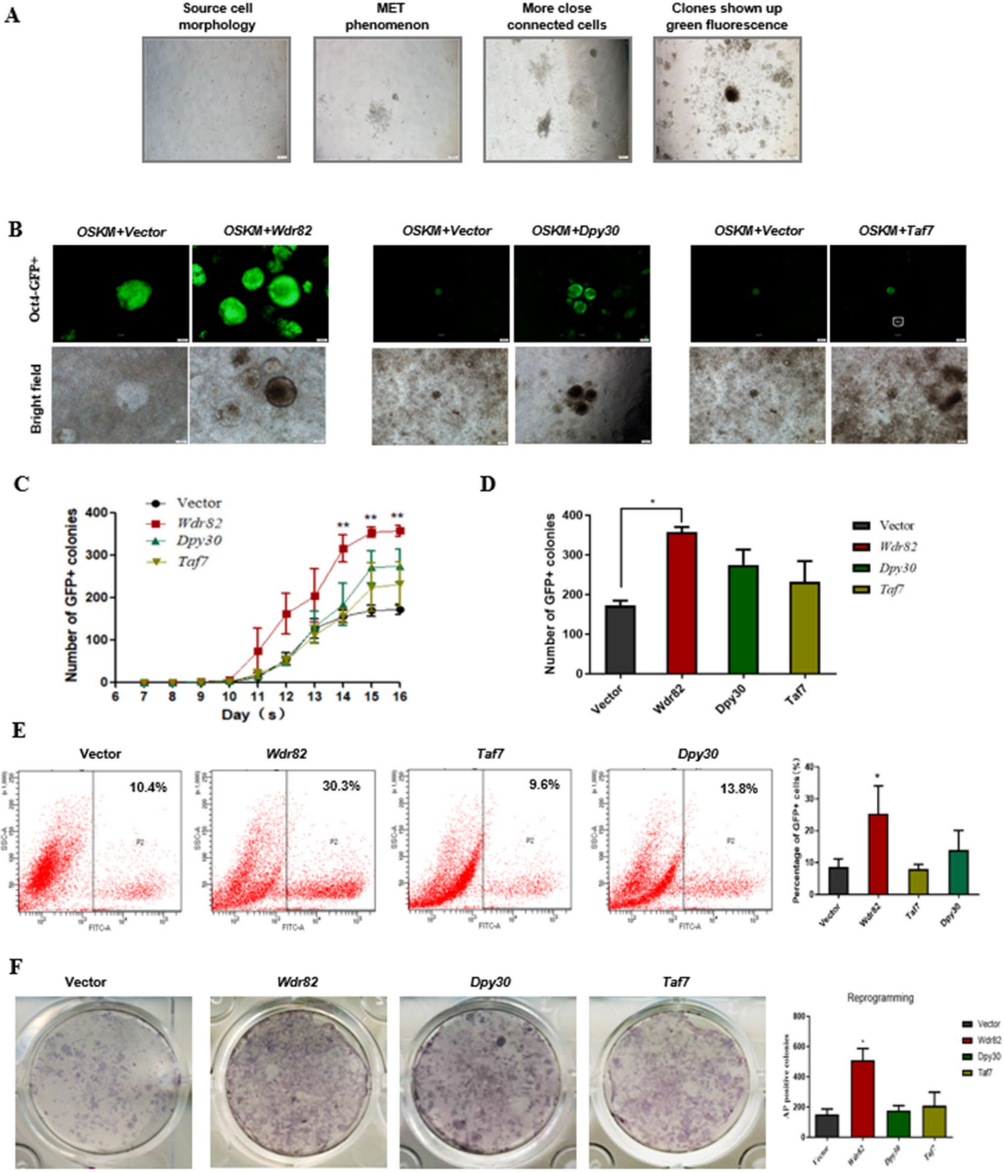
Overexpressed Wdr82 increases reprogramming efficiency. **A** Changes in the morphology of cells during somatic cell reprogramming as observed with bright field microscopy. **B** The morphology of the clones overexpressing Wdr82, Dpy30, or Taf7 as observed by bright field and fluorescence microscopy. **C** and **D** In the reprogramming experiment, overexpressed Wdr82 led to the production of the most GFP^+^ clones, as shown in the line graph (**C**) and the histogram (**D**). **E** Among the three analyzed factors, by the end of the reprogramming induction period, Wdr82 overexpression produced the highest proportion of Oct4-positive cells, as assessed by flow cytometry. **F** Among the three factors, Wdr82 overexpression induced the greatest production of AP^+^ clones at the end of the reprogramming induction period. The number of positive clones is indicated as the mean ± standard error, *n = *3; significant difference was determined by t test: *P < 0.05, **P < 0.01

Since MII oocyte-derived transcription factors might increase the generation of induced pluripotent stem cells [12, 35], three highly expressed factors in MII oocytes that possibly participating in epigenetic modification of Chromatin were selected for analysis, namely, *Wdr82* (WD repeat domain 82), *Dpy30* (dpy-30 histone methyltransferase complex regulatory subunit), and *Taf7* (TATA-box binding protein-associated factor 7), and their fusion expression clones were inserted into a Fuw-TetOn vector backbone. *Oct4*-GFP/Rosa26-M2rtTA mouse embryonic fibroblasts (OR-MEFs) were used as the sources of somatic cells to induce pluripotent stem cell generation at the basis of the induction by Yamanaka factors (OSKM factors) (Figs. S1, 1A). The effects of these three factors on somatic cell reprogramming were evaluated. The results indicated that among the three factors, *Wdr82* overexpression resulted in the most significant promotion of somatic cell reprogramming, and its effects were very stable. Many more *Oct4*-GFP^+^ clones were produced during reprogramming induced by OSKM + *Wdr82* when compared with that induced with the OSKM control and/or by the addition of *Dpy30*/*Taf7* (Fig. 1B). Moreover, the number of *Oct4*-GFP^+^ clones was significantly increased, by 2.1-fold, as compared to that of the OSKM control clones (Fig. 1C and D). These data were confirmed via flow cytometry analysis, which showed that the increase in *Oct4*-GFP^+^ clones was 2.9-fold higher than that in the control clones (Fig. 1E). In addition, alkaline phosphatase (AP) staining showed that the number of AP^+^ clones increased by 3.4-fold (Fig. 1F). These findings indicate that *Wdr82* increased somatic cell reprogramming efficiency. Then, the pluripotency of the reprogrammed *Oct4*-GFP^+^ clones was further assessed.

### OSKM + *Wdr82* reprogrammed cell lines showed pluripotency and chimeric mice were produced

**Fig. 2:**
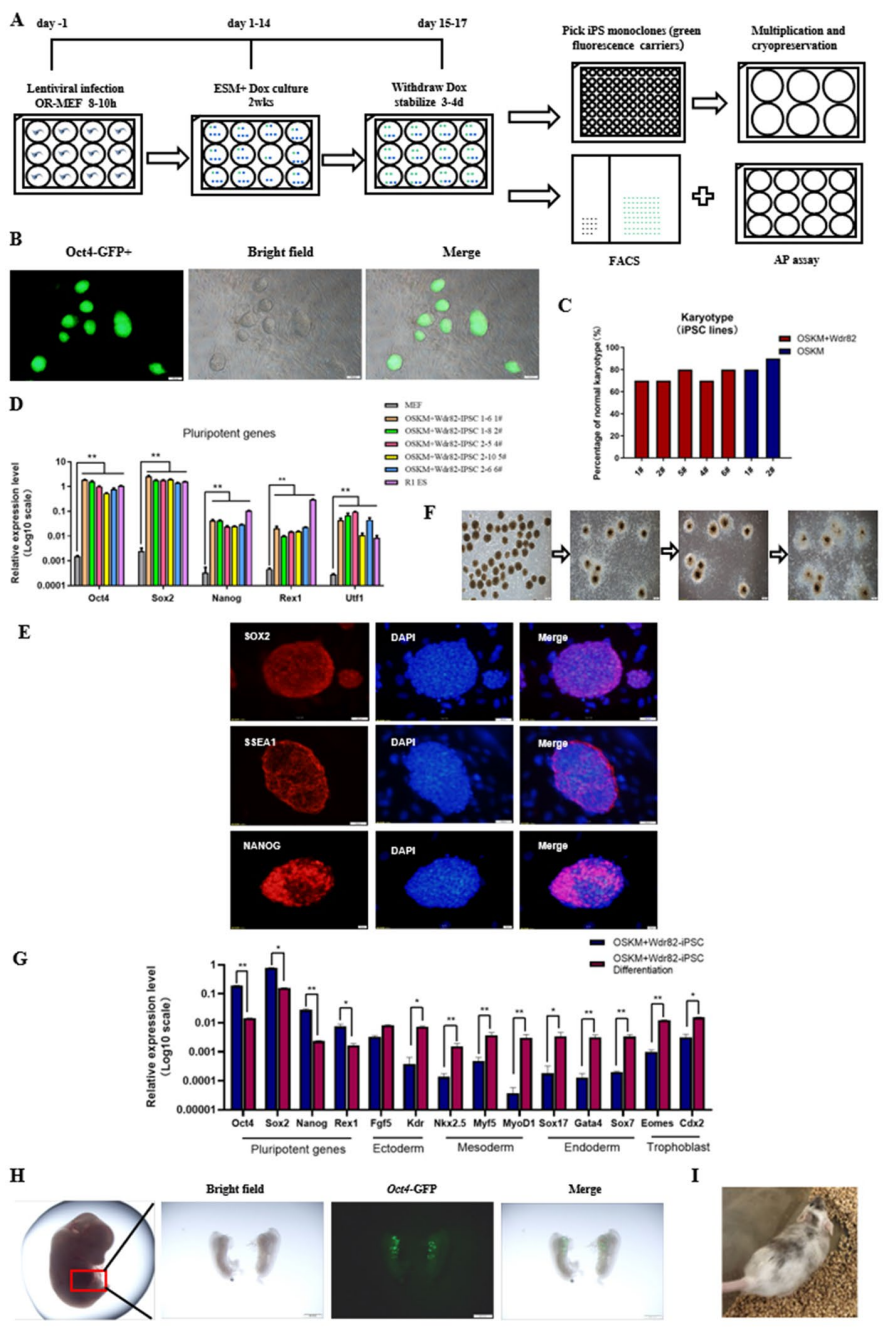
Establishment and function verification of the OSKM + Wdr82-iPSC line. **A** The flow chart indicates the establishment and verification of the OSKM + Wdr82-iPSC line. **B** OSKM + Wdr82-iPS cell morphology in bright and dark fields. **C** Karyotype evaluation: The correct karyotype acquisition rate of OSKM + Wdr82-iPSC line was equivalent to that of the OSKM control cells, detailed in Fig. S2; **D** RT-PCR detection of pluripotency gene (Oct4, Sox2, Nanog, Rex1 and Utf1) expression: The expression levels of these genes in the OSKM + Wdr82-iPSC line were equivalent to that in ESCs (positive controls) and much higher than that in MEFs (negative controls). The fold change in expression is indicated on a log10 scale. **E** Immunofluorescence staining for detection of pluripotency proteins (SOX2, SSEA1, and NANOG) in the OSKM + Wdr82-iPS cell line. **F** In vitro EB differentiation of the OSKM + Wdr82-iPSC line. **G** RT-PCR measurement of the expression of differentiated marker genes. The differentiated OSKM + Wdr82-iPSCs showed higher expression levels of marker genes that were specifically expressed in the three embryonic germ layers, as indicated, and lower expression levels of pluripotency genes than those in the parent iPSCs. The expression fold change is indicated on a log10 scale. **H** In vivo chimerism experiment: The Oct4-GFP derived from OSKM + Wdr82-iPSC line can be observable in the genital crest, indicating the obtained chimeras mice. **I** Representative image of adult chimeric mice from two OSKM + Wdr82*-*iPSC lines, with black and white coat color. * *P* < 0.05 and ** *P* < 0.01

To verify the promoting effect of *Wdr82* on mouse somatic cell reprogramming, in this study, *Oct4*-GFP^+^ monoclonal cells obtained by reprogramming were selected (denoted as OSKM + *Wdr82*-iPSCs; Fig. 2A), and then, the pluripotency and differentiation potential of these cells were evaluated. The results showed that the morphology of the OSKM + *Wdr82*-iPSCs was similar to that of ESCs and that the OSKM + *Wdr82*-iPSCs expressed *Oct4* (a fluorescent reporter gene) (Fig. 2B) and presented a normal karyotype (Fig. S2, Fig. 2C). The OSKM + *Wdr82*-iPSC line with the correct karyotype was selected for pluripotency assessment. The RNA expression levels of the pluripotent genes *Oct4*, *Sox2*, *Nanog*, *Rex1* and *Utf1* in the OSKM + *Wdr82*-iPSC line were comparable to those in ESCs and markedly higher than those in MEFs (Fig. 2D), and the protein expression of the pluripotent markers SOX2, NANOG, and SSEA1 was evident (Fig. 2E). An in vitro embryoid body (EB) differentiation experiment performed via the hanging drop method (Fig. 2F) indicated that the expression of the pluripotency marker genes specific to the germ layers of differentiated EB spheres was markedly enhanced (Fig. 2G), suggesting that the OSKM + *Wdr82*-iPSC line showed the potential to differentiate into three embryonic germ layers in vitro*.* A fundamental in vitro chimeric mouse experiment confirmed that the OSKM + *Wdr82*-iPSC line transmitted genetic material to offspring mouse cells, as indicated by the embryonic genital ridge in the offspring mice expressing the *Oct4*-GFP gene (Fig. 2H). An assessment of the adult chimeric mice (Figs. 2I and S3) validated that the OSKM + *Wdr82*-iPSC line showed pluripotency.

In addition, although it promoted somatic cell reprogramming, *Wdr82* did not replace any of the four Yamanaka factor genes. To further study the role of *Wdr82* in reprogramming induction, a series of induction experiments was performed by replacing each of the OSKM factors with *Wdr82*, denoted as OSKM, OSK, OSM, OKM and SKM factors, with or without the addition of *Wdr82*. The results of three biological replicate experiments showed that cells carrying Wdr82 and the four replacement genes expressed *Oct4*-GFP^+^ clones in numbers are comparable to the corresponding negative control cells (without *Wdr82* overexpression) but in significantly lower numbers than the positive control OSKM cells (Fig. S4A and S4D), suggesting that *Wdr82* did not replace the function of the genes encoding of any of the four Yamanaka factors. This result was confirmed by flow cytometric analysis and alkaline phosphatase activity assay carried out at the end of the reprogramming induction period (Fig. S4B and S4C). In summary, *Wdr82* cannot be considered a substitute for any of the four Yamanaka factors, although *Wdr82* promotes somatic cell reprogramming.

### Expression inhibition of *Wdr82* significantly suppresses reprogramming efficiency

**Fig. 3:**
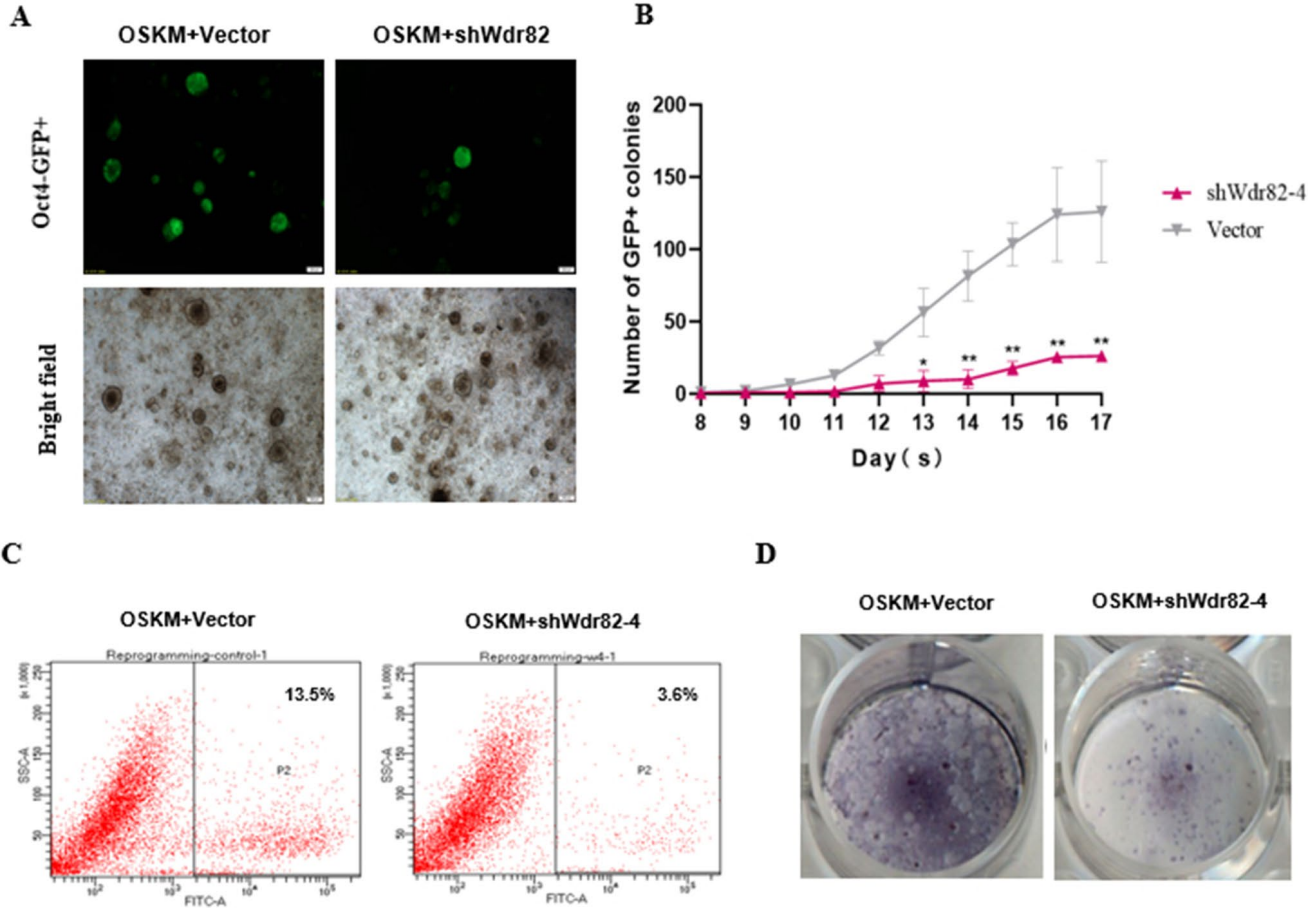
Knocking down Wdr82 inhibits somatic cell reprogramming. **A** The morphology of OSKM + shWdr82 (knockdown) reprogrammed cells and the OSKM control cells. **B** When compared with the number among OSKM control cells, knocking down Wdr82 (OSKM + shWdr82) significantly reduced the number of Oct4-positive clones. **C** The proportion of Oct4 + cells after Wdr82 was knocked down was significantly reduced compared with that in the OSKM control cell group. **D** When compared with that of the OSKM control cells, the AP^+^ clone number was significantly reduced. * *P* < 0.05 and ** *P* < 0.01

To confirm the effect of *Wdr82* on the reprogramming efficiency, a reprogramming induction experiment in cells which *Wdr82* was knocked down via *Wdr82*-specific short hairpin RNA (shRNA) plasmids was conducted. The results showed that when compared with the OSKM control groups, all three corresponding W*dr82-*knockdown constructs significantly inhibited reprogramming efficiency (Figs. S5A, 3A) in three replicate experiments. Moreover, the number of *Oct4*-GFP^+^ clones after *Wdr82* knockdown was markedly reduced through the reprogramming period, with the inhibitory reprogramming efficiency induced by sh*Wdr82*-4 being particularly notable (Figs. 3B and C, S5B). These findings were confirmed via both flow cytometry and AP staining analysis (Figs. 3D, S5C and S5D). In general, knocking down *Wdr82* significantly inhibited somatic cell reprogramming efficiency.

### The promoting effect of *Wdr82* on reprogramming efficiency was mediated by inhibiting OXPHOS activity, suggesting a regulatory role in cellular energy metabolism.

**Fig. 4:**
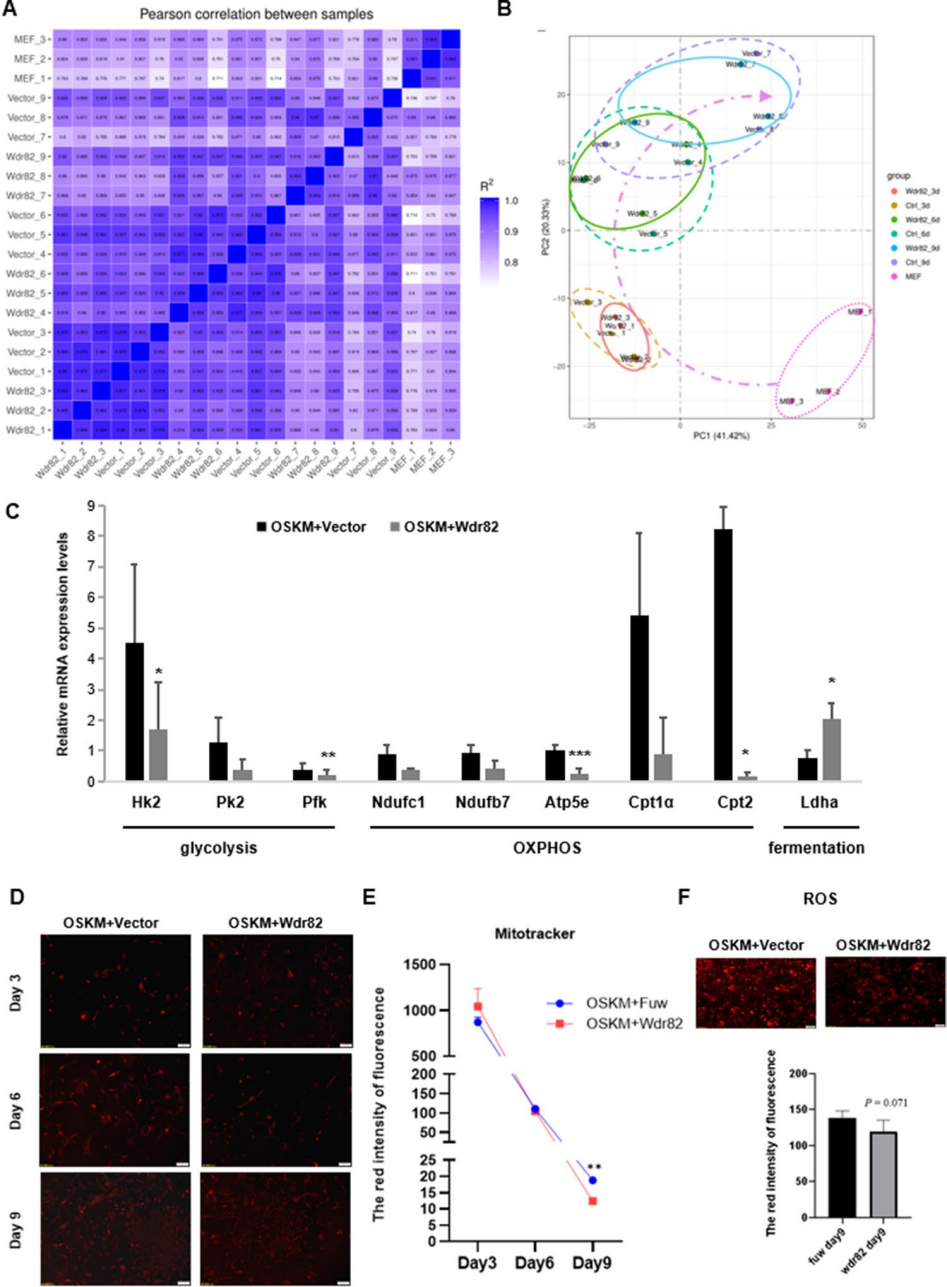
Transcriptome analysis of OSKM + Wdr82 cells during reprogramming induction suggested that Wdr82 promotes somatic cell reprogramming by inhibiting OXPHOS. **A** Heatmap showing the correlation coefficients among samples; Vector-1, Vector-2, Vector-3, ad Wdr82-1, Wdr82-2, and Wdr82-3 represent cell samples collected from the OSKM and OSKM + Wdr82 groups, respectively, on reprogramming Day 3. Similarly, Vector-4, Vector-5, Vector-6, Wdr82-4, Wdr82-5, and Wdr82-6 samples were obtained on reprogramming Day 6, and Vector-7, Vector-8, Vector-9, Wdr82-7, Wdr82-8, and Wdr82-9 samples were collected on Day 9. MEF-1, MEF-2, and MEF-3 were used as the negative controls. **B** Principal component analysis results showing that the expression pattern in the MEFs was markedly different from that of the cells reprogrammed for 3, 6 and/or 9 days. In the reprogrammed cells, the expression patterns in both the OSKM and OSKM + Wdr82 groups gradually changed throughout the reprogramming process, following an upward sloping curve. Samples in the enclosed solid and dotted line represent samples from OSKM + Wdr82 (Wdr82) and OSKM (Ctrl) cells, respectively. **C** The expression of genes involved in glycolysis (*Hk2*, *Pk2*, and *Pfk*) and OXPHOS (*Ndufc1*, *Ndufb7*, *Atp5e*, *Cpt1α*, and *Cpt2*) in addition to that of Wdr82 was inhibited during reprogramming (Day 6 ~ Day 9), while fermentation gene expression associated with induced lactate production (*Ldhα*) was significantly enhanced as compared to that in the OSKM-only control group. **D** A MitoTracker assay indicated that the number of mitochondria in cells after the addition of Wdr82 slowly decreased compared to that in the OSKM control group during cell reprogramming (Day 3 ~ Day 9). Red fluorescence represents mitochondria in the cytoplasm. **E** Quantitation of the MitoTracker assay data. **F** ROS production in reprogrammed cells on Day 9 was decreased after the addition of Wdr82, and the quantification results were shown as in the column chart. * *P* < 0.05, ** *P* < 0.01 and *** *P* < 0.001

To explore the biological pathways by which *Wdr82* increases reprogramming efficiency, a high-throughput transcriptome sequencing (RNA-seq) experiment was performed during the reprogramming process on Days 3, 6 and 9, with corresponding negative control cells (no Wdr82 overexpression) and source cell MEFs included. A heatmap of the Pearson correlation coefficients indicated that the three MEF samples showed completely different RNA expression patterns and that the three biological replicates of each reprogramming sample showed reliable and closely associated variable clusters (Fig. 4A). The results of a principal component analysis (PCA) suggested that the gene expression patterns in reprogrammed cells gradually changed to follow a sloping curve and that, compared to the respective control cells (OSKM + Vector-N), the reprogrammed cells with *Wdr82* (OSKM + Wdr82-N) were more concentrated in clusters (solid-lined ovals) (Fig. 4B). The differentially expressed genes (up- and downregulated) on reprogramming Day 9 were much more abundant than those on Day 6 and/or Day 3 (Fig. S6A), which was largely consistent with the morphological observations made during cell reprogramming for approximately 9 days, which showed an increase in colony numbers and green fluorescence. To explore the gene function and biological pathways of *Wdr82* in reprogramming, a joint GO and KEGG analysis was conducted based on the differentially expressed genes identified on reprogramming Day 9, and the results indicated that these genes were mainly enriched in oxidative phosphorylation (OXPHOS) and ribosomes (Fig. S6B). Moreover, two biological processes were enriched as determined via an analysis of the protein−protein interaction network (Fig. S6C). The expression of genes involved in oxidative phosphorylation was validated by quantitative RT-PCR measurements; these genes included *Ndufas*, *Ndufbs*, *Coxs* and/or *Atpases*, were significantly suppressed on reprogramming Day 9 when compared with their expression in the OSKM control cells without *Wdr82* addition (Fig. S6D).

**Fig. 5:**
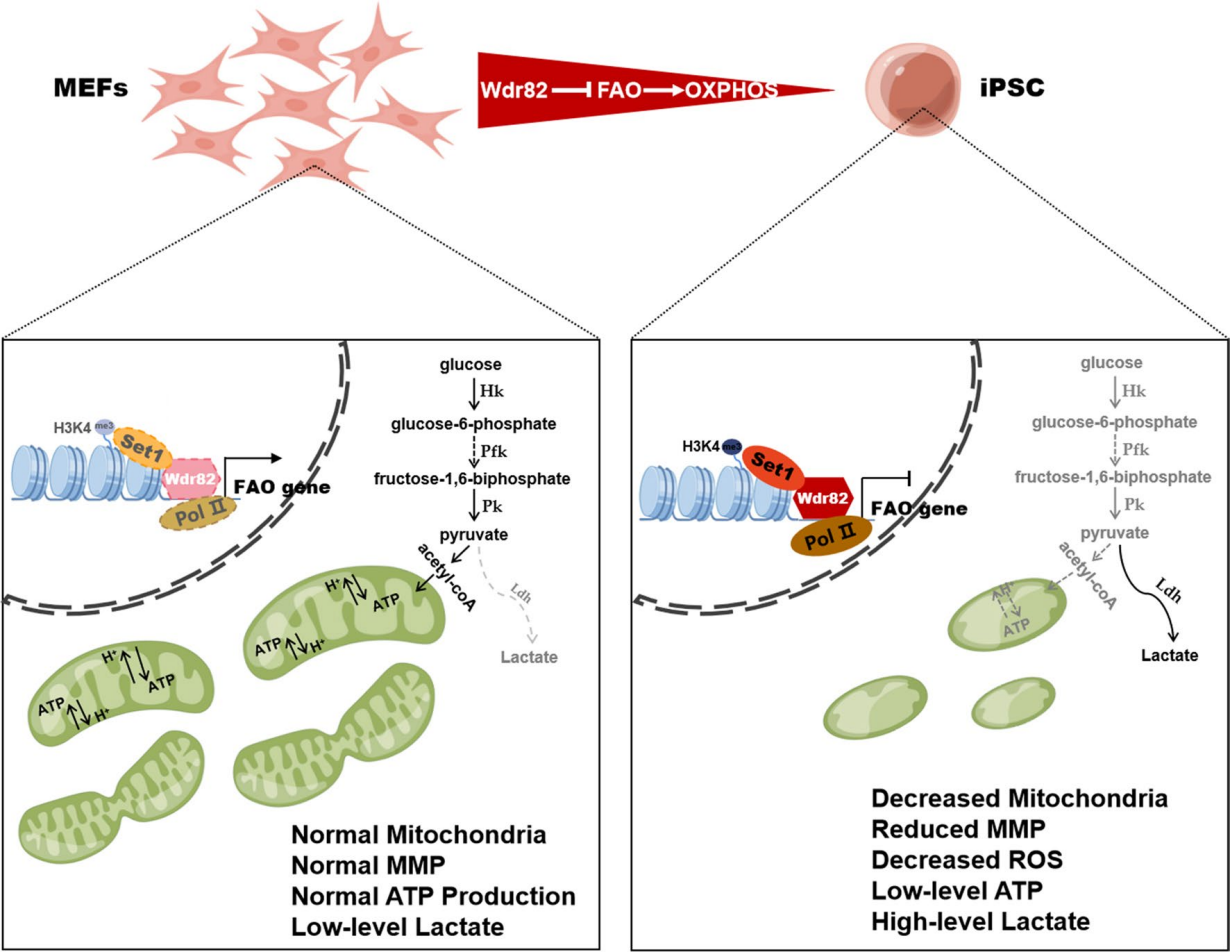
Abundantly expressed factor Wdr82 in oocytes can facilitate iPSCs generation by inhibiting mitochondrial oxidative phosphorylation. Wdr82, a C-terminal domain-binding protein that recruits the Setd1A/B histone H3K4 methyltransferase complex and phosphorylates RNA polymerase II, suppresses the transcrptions of genes (*Nudf* etc.) involved in OXPHOS in mitochondria in the early stage of the reprogramming, thereby inhibiting OXPHOS and transferring the basis of the carbon energy source from FAO to glycolysis (lactate), promoting fermentation, and ultimately mediating cell fate reprogramming. The process is accompanied with changes of mitochondrial quantity and structure. The study provides evidence showing that metabolic switching mediated through metabolic function, epigenetic modifications and/or gene expression is critical for cell fate determination

This finding suggested that in the cell reprogramming process, the energy metabolism pathway was specifically reprogrammed, as indicated by the significant inhibition of OXPHOS and aerobic metabolism in the reprogrammed cells. Therefore, a series of experiments to assess the energy metabolism pathway during somatic reprogramming was carried out. First, in addition to that of the genes involved in OXPHOS (*Ndufc1*, *Ndufb7*, *Atp5e*, *Cpt1α*, and *Cpt2*), the expression of genes involved in glycolysis (*Hk2*, *Pk2*, and *Pfk*) was inhibited during reprogramming (Day 6–Day 9, Fig. 4C). In contrast, the expression of the gene involved in fermentation-inducing lactate production (*Ldhα*) was significantly enhanced. Second, a MitoTracker assay indicated that the number of mitochondria within the cell after the addition of *Wdr82* was slowly decreased as compared to that in the OSKM control cells during cell reprogramming (Day 3–Day 9, Fig. 4D and E). Third, a JC-1 mitochondrial membrane potential assay indicated that cells treated with Wdr82 + OSKM showed significantly more unhealthy/damaged mitochondria than those treated with OSKM only during the reprogramming period, especially on Day 9 (Figure S6E and S6F). Moreover, gradually decreased ROS accumulation was accompanied by slowly inhibited mitochondrial function (Fig. 4F) in the cells undergoing reprogramming (Day 9). All of the aforementioned evidence suggested that *Wdr82* promotes somatic cell reprogramming by inhibiting OXPHOS in mitochondria but enhancing fermentation (Fig. 5). The study provides a link between mitochondrion metabolism and cell reprogramming efficiency.

## Discussion

In this study, three highly expressed factors in MII phase oocytes, *Wdr82*, *Dpy30* and *Taf7*, were selected as possible candidates for promoting somatic cell reprogramming. Our data showed higher reprogramming efficiency after the addition of Wdr82 than that of the four Yamanaka factors only (Fig. 1), providing scientific evidence for the hypothesis that abundantly expressed oocyte factors promote the generation of induced pluripotent stem cells and/or embryonic development [12, 35]. In particular, iPSC quality after the addition of *Wdr82* was shown to be excellent, with pluripotency and heritability to chimeric offspring mice (Fig. 2).

In terms of the molecular mechanisms underlying how oocyte factors regulate the reprogramming process may differ depending on the factor. Our investigation revealed that Wdr82 promoted programming efficiency via OXPHOS inhibition at the early stage of programming (Fig. 4). Meanwhile, the addition of Wdr82 did enhanced the H3K4me3 level in reprogrammed cells at day 6 and day 9 (Fig. S7), confirming its recruitment role in the Setd1A/B complex, a H3K4me3 methyltransferase complex, during the reprogramming process. Moreover, Wdr82 failed to be a suitable replacement for any of the four Yamanaka factors (Fig. S4), suggesting its unique roles in the regulation of cell fate determination. With regard to OXPHOS during reprogramming, increasing evidence has suggested that a cell fate transition is usually accompanied by reconstruction of the energy metabolism pathway. The metabolism of somatic cells and stem cells is very different. As the main sites of cell respiration and energy production, mitochondria play important roles in cellular energy metabolism. Somatic cells heavily rely on mitochondrial oxidative phosphorylation as the main source of energy [9], while ESCs rely on glycolysis for ATP production-based energy [7]. The upregulation of glycolytic enzymes and the downregulation of electron transport chain subunits switch mitochondrial oxidative metabolism into a glycolysis-dependent state, which is the basis for acquiring a pluripotent phenotype (Fig. 5) [11, 34, 40]. For example, the histone acetyltransferase MOF directly activates the fatty acid oxidation (FAO) pathway in mitochondria, thereby blocking quiescence in ESCs, which show ground state pluripotency [20]. Similarly, a protein on the surface of lipid droplets (LDs), perilipin 2 (Plin2), accelerates ESC decline in pluripotency by enhancing lipidomic remodeling and histone acetylation [39], indicating a mechanism linking LD homeostasis to mitochondrial remodeling (the development of mitochondrial cristae and FAO) and epigenetic regulation [26–28]. FAO provides the carbon source for fueling mitochondrial respiration, that is, OXPHOS. In summary, the importance of energy metabolism is not only essential for cell survival and proliferation, but is also increasingly recognized in pluripotency maintenance and cell fate determination [26, 29]. Here, we found that *Wdr82* directly modifies the expression of genes involved in mitochondrial OXPHOS, thereby transferring the basis of the carbon energy source from FAO to glycolysis (lactate), inhibiting OXPHOS, promoting fermentation (Fig. 4), and ultimately mediating cell fate reprogramming (Fig. 5).

Moreover, *Wdr82*, a scaffold protein, is a component of the Setd1A/B complex, a H3K4me3 methyltransferase complex related to gene transcription activation, that regulates the expression of many genes in early embryonic development. Studies have reported that knocking down *Wdr82* in mESCs significantly reduced the level of H3K4me3, slows the cell proliferation rate and inhibits cell cycle progression through the p53−p21 pathway [3]. The loss of *Wdr82* specificity leads to a significant decrease in the rate of H3K4 trimethylation and the level of actively transcribed genes in chromatin [38]. *Oct4* is a downstream factor of the Setd1A/B complex, and the absence of *Wdr82* reduces the Setd1A/B complex level, which hinders the growth and development of an embryo, in some cases leading to embryonic death [3]. *Wdr82* is also present in the protein phosphatase 1 (PP1) complex. The PP1 nuclear targeting subunit (Pnuts) is necessary for PP1 recruitment to active transcription sites, and it dephosphorylates the carboxy-terminal repeat domain of RNA polymerase II (Pol II) at active transcription sites [1]. *Wdr82* inhibits transcription−replication conflict by promoting the degradation of Pol II on chromatin and reducing its residence time. Pnut-PP1-mediated dephosphorylation of the carboxy-terminal domain of Pol II led to results similar to *Wdr82* [22]. In summary, *Wdr82* activates the transcription of abundant downstream genes, which may be associated with metabolic functions, epigenetic modification and/or gene repression, by recruiting transcription factors that bind to Pol II.

Knocking down *Wdr82* significantly decreased the H3K4 trimethylation rate in mESCs, causing considerable delays in the G1 phase and a slowed proliferation rate. The expression of the cell cycle-dependent kinase inhibitor *p21* and tumor suppressor gene *p53* was significantly upregulated, indicating that inhibition of the cell cycle by *Wdr82* knockdown was realized through the p53−p21 pathway. Dai et al. reported that Akt directly phosphorylates *Oct4* to regulate the formation of *Oct4/Sox2* heterodimers and promotes p300-mediated acetylation of *Oct4, Sox2* and *Klf4*, thereby indirectly promoting iPSC formation [8]. P53 can directly control cell death and mitochondrial respiration and can act on the *Mdm2, Mdm4, Arf* and *Bmi1* factors in mitochondria. Mdm4 is related to the control of cell death. In summary, the p53 pathway is closely related to mitochondrial function and fine tunes metabolic processes [21]. Akt phosphorylates *Mdm2* and promotes the degradation of p53 by the proteasome [32], and the caspase cascade induced by p53 accelerates the degradation of *Mdm2* and Akt [13]. Therefore, *Wdr82* is thought to exert its regulatory effect via the Pi3k−Akt pathway, thereby inhibiting the p53 signaling pathway. *P21*, a target gene downstream of p53, inhibits the expression of genes associated with mitochondrial oxidative phosphorylation and possibly that of epigenetic modifications and/or pluripotent genes.

Recent evidence supports the idea that mitochondria are signaling organelles that dictate stem cell fate and function. Mitochondrial metabolism is linked to the TCA cycle/ATP production and aerobic metabolism to support cell fate determination through a wide variety in regulatory cellular functions [4]. Moreover, cell fate decisions are susceptible to intrinsic metabolic bias imposed by selectively inherited mitochondria [10, 26]. Moreover, lactate, as a byproduct of glycolysis, which is upregulated in response to hypoxia (anaerobic metabolism), has been identified by researchers of the Warburg effect and has recently been considered the primary substrate of lactylation, an important posttranslational modification, thereby playing a critical role in regulating homeostatic and pathological processes [26–28, 41]. Therapeutics based on mitochondrion metabolism remodeling may be attractive strategies to improve stem cell function to attenuate disease and/or promote healthy aging due to their fate-determining chromatin epigenetic modifications, hypoxic transcriptional responses and/or immune functions [2, 30, 42].

## Conclusion

Overall, a transcription factor abundantly expressed in MII oocytes*, Wdr82,* can increase the efficiency of somatic cell reprogramming induction. A *Wdr82*-expressing iPSC line showed pluripotency with the potential to differentiate into three germ layers and to transmit genetic material to chimeric offspring. The significant suppression of OXPHOS in mitochondria underlies the molecular mechanism by which *Wdr82* promotes the efficiency of somatic cell reprogramming. Our study provides a link between mitochondrion metabolism and cell fate determination.

## Supplementary Information

Below is the link to the electronic supplementary material.

Supplementary file1 (DOCX 809 KB)

## Data Availability

The RNA-seq data used to support the findings of this study are deposited in GEO/NCBI and publicly available with ID of PRJNA975608.

## Abbreviations

AP: Alkaline phosphatase
ATP: Adenosine triphosphate
Atp5e: ATP synthase H + transporting mitochondrial F1 complex epsilon subunit
c-Myc: MYC proto-oncogene, a bHLH transcription factor
Cpt1α: Carnitine O-palmitoyltransferase 1
Cpt2: Carnitine O-palmitoyltransferase 2
Dpy30: Dpy-30 histone methyltransferase complex regulatory subunit
FAO: Fatty acid oxidation
Glis1: GLIS family zinc finger 1
H1foo: Oocyte-specific H1 histone
Hk: Hexokinase
iPSC: Induced pluripotent stem cell
Klf4: Kruppel like factor 4
Mdm2/4: MDM2/4 proto-oncogene
MEF: Mouse embryonic fibroblast
MMP: Mitochondrial membrane potential
Nanog: Nanog homeobox
Nduf: NADH dehydrogenase (ubiquinone) 1 subunit, mitochondrial
Obox1: Oocyte-specific homeobox 1
Oct4: POU domain, class 5, transcription factor 1
OSKM: Oct4, Sox2, Klf4 and c-Myc
OXPHOS: Oxidative phosphorylation
Pf: Pyruvate kinase
Pfk: ATP-dependent 6-phosphofructokinase
Rex1: RNA exonuclease 1
ROS: Reactive oxygen species
Setd1b: SET domain-containing 1B, histone lysine methyltransferase
Sox2: SRY-box transcription factor 2
Taf7: TATA-box-binding protein associated factor 7
TCA cycle: Tricarboxylic acid cycle
Tcl1: T-cell lymphoma breakpoint 1
Tet1: Tet methylcytosine dioxygenase 1
Utf1: Undifferentiated embryonic cell transcription factor 1
Wdr5: WD repeat domain 5
Wdr82: WD repeat domain 82
Yamanaka factors: Oct4, Sox2, Klf4 and c-Myc
Zscan4: Zinc finger and SCAN domain containing 4
