# Supplementary material for: WD repeat domain 82 (Wdr82) facilitates mouse iPSCs generation by interfering mitochondrial oxidative phosphorylation and glycolysis
Source: Cell Mol Life Sci. 2023 Jul 20;80(8):218. doi: 10.1007/s00018-023-04871-z (PMC10359378; doi:10.1007/s00018-023-04871-z)
Supplement: Supplementary file 1 — Supplementary file1 (DOCX 809 KB) [file 18_2023_4871_MOESM1_ESM.docx]

**WD Repeat Domain 82 (Wdr 82) Facilitates Mouse iPSCs Generation by Interfering Mitochondrial Oxidative Phosphorylation and Glycolysis**

Guina Cui^1,2,3^, Jingxuan Zhou^1,3^, Jiatong Sun^1,2^, Xiaochen Kou^2^, Zhongqu Su^1,2^, Yiliang Xu^1,2^, Tingjun Liu^1^, Lili Sun^1^, Wenhui Li^1^, Xuanning Wu^1^, Qingqing Wei^1^, Shaorong Gao^2,4*^，Kerong Shi^1*^


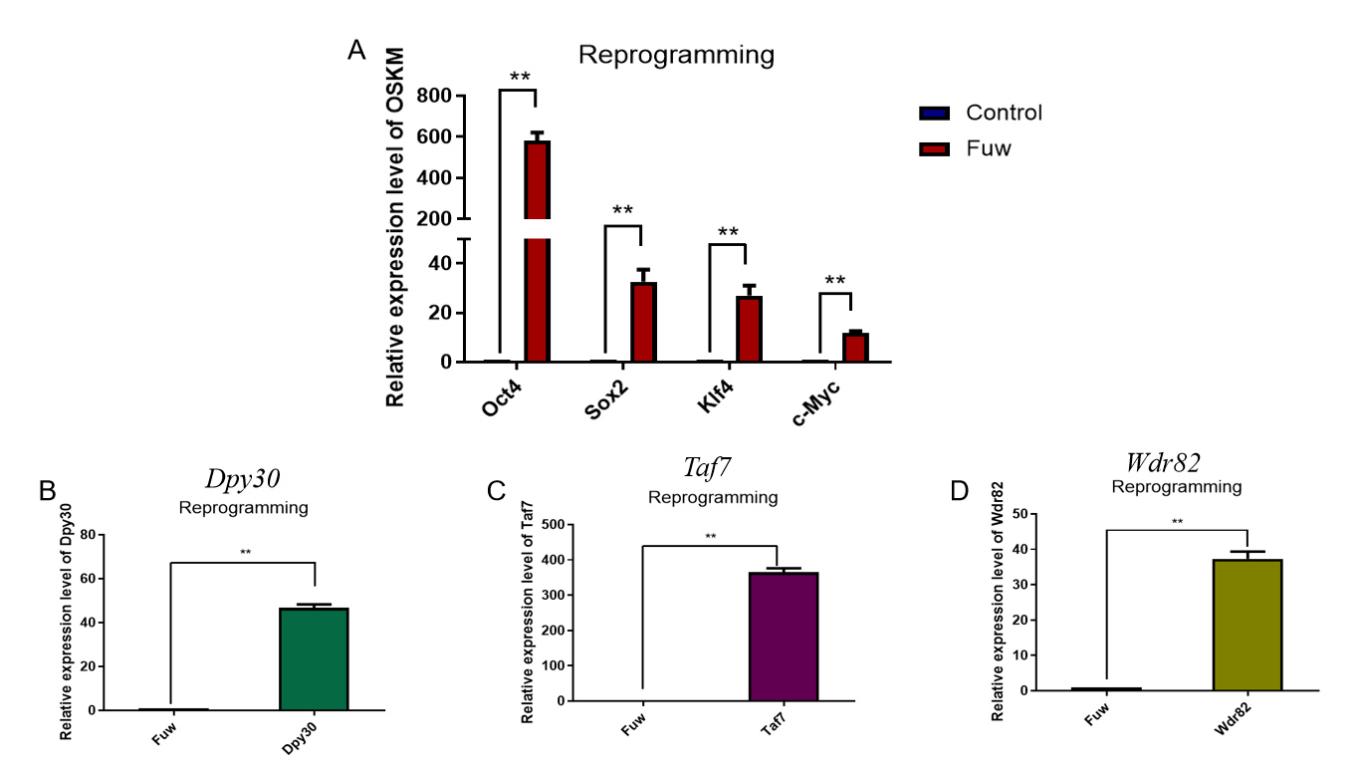


**Figure S1**. Yamanaka factors (*Oct4*, *Sox2*, *Klf4*, *c-Myc*, shown in panel A) and/or the three target factors (*Dpy30* in panel B, *Taf7* in panel C or *Wdr82* in panel D) were detected to be overexpressing in cells at reprogramming Day 2 via RT-PCR method. * *P* <0.05 and ** *P* <0.01.


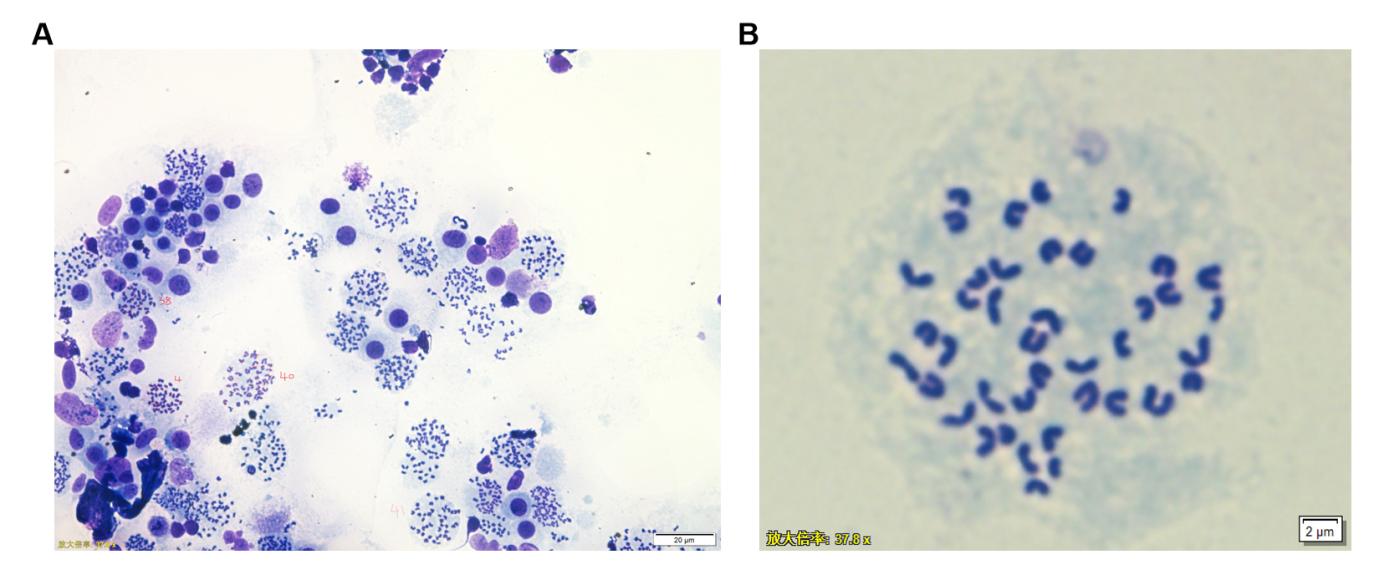


**Figure S2.** Reprsentative karyotype images of OSKM+*Wdr82*-iPS cell line, showing multiple cell metaphases with different number of chromosomes at both lower (A) and higher (B) magnifications, which indicates that the induced iPS clones have correct karyotype.


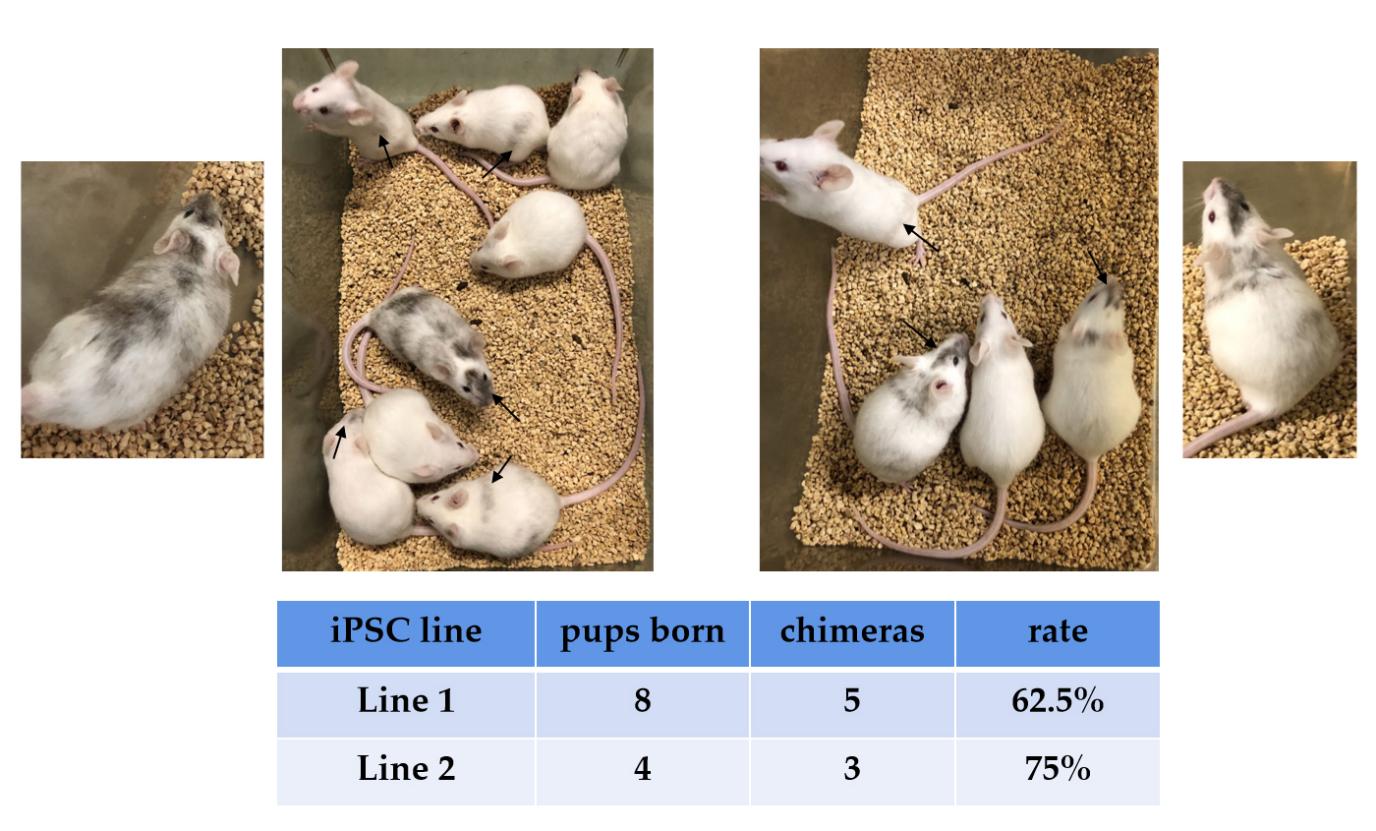


**Figure S3.** Representative images of adult chimeric mice (black arrows) from two OSKM+*Wdr82-*iPS cell lines (upper panel) and the corresponding rate of chimera formation (lower panel). The degree of chimerism can be evaluated by the white and black coat color.


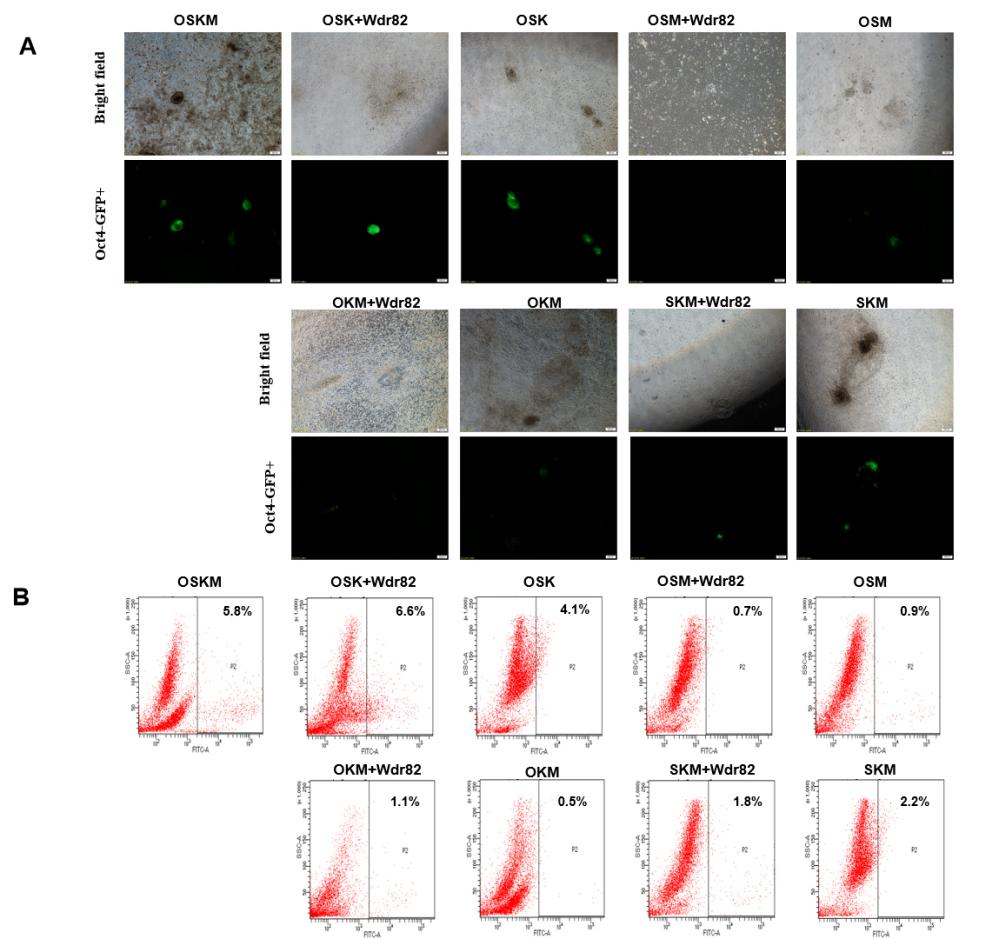


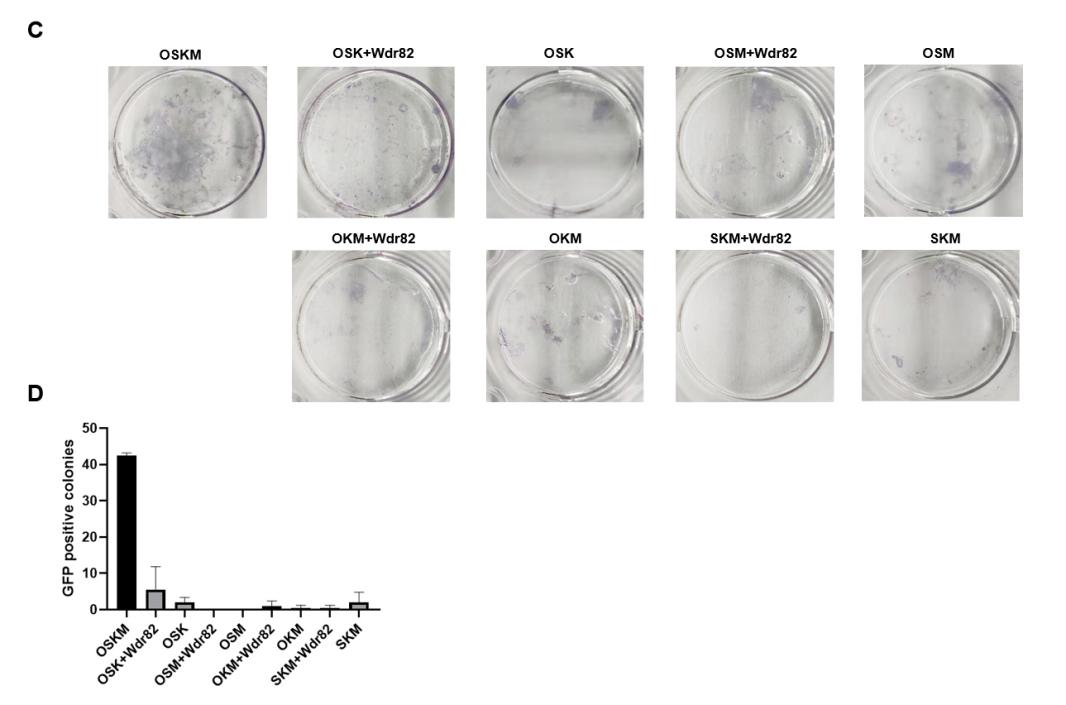


**Figure S4. Wdr82 single-factor replacement reprogramming experiment**

A. Clonal morphology under bright and fluorescence field upon Wdr82 replacement of each one of the four-factors, as well as OSKM control. The combination of kept factors with Wdr82 are indicated. B. Flow cytometric analysis of obtained Oct4+ cell percentage in each combination during reprogramming induction. C. AP+ staining results of each combination during reprogramming induction. D. Statistics of the number of Oct4-GFP+ clones during reprogramming induction.


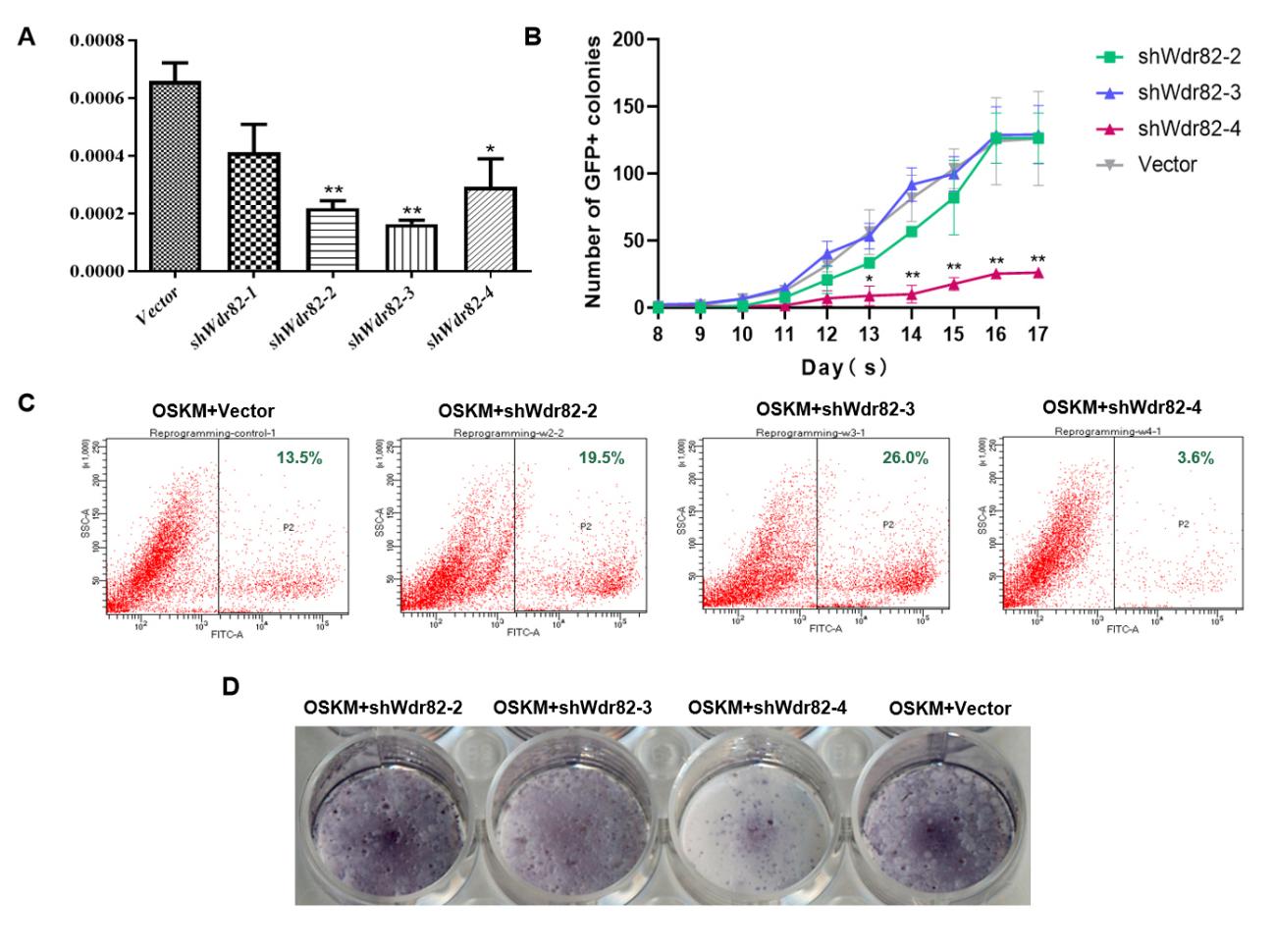


**Figure S5. Knockdown of Wdr82 inhibits OSKM mediated reprogramming efficiency.** A. The assessment of Wdr82 knockdown efficiency via RT-PCR method, showing that constructs of shWdr82-2, shWdr82-3 and shWdr82-4 had significant knockdown efficiency, good to use for their follwing assessments on reprogramming efficiency. B. Compared with the OSKM control (Vector), knockdown of Wdr82 (OSKM+shWdr82-4) significantly reduced the number of Oct4+ clones; C. The proportion of Oct4+ cells upon Wdr82 knockdown (OSKM+Wdr82-4) was significantly reduced, compared with the OSKM control. D. Compared with the OSKM control, AP+ clones number upon knockdown of Wdr82 (OSKM+Wdr82-4) is significantly reduced. * *P* <0.05 and ** *P* <0.01.


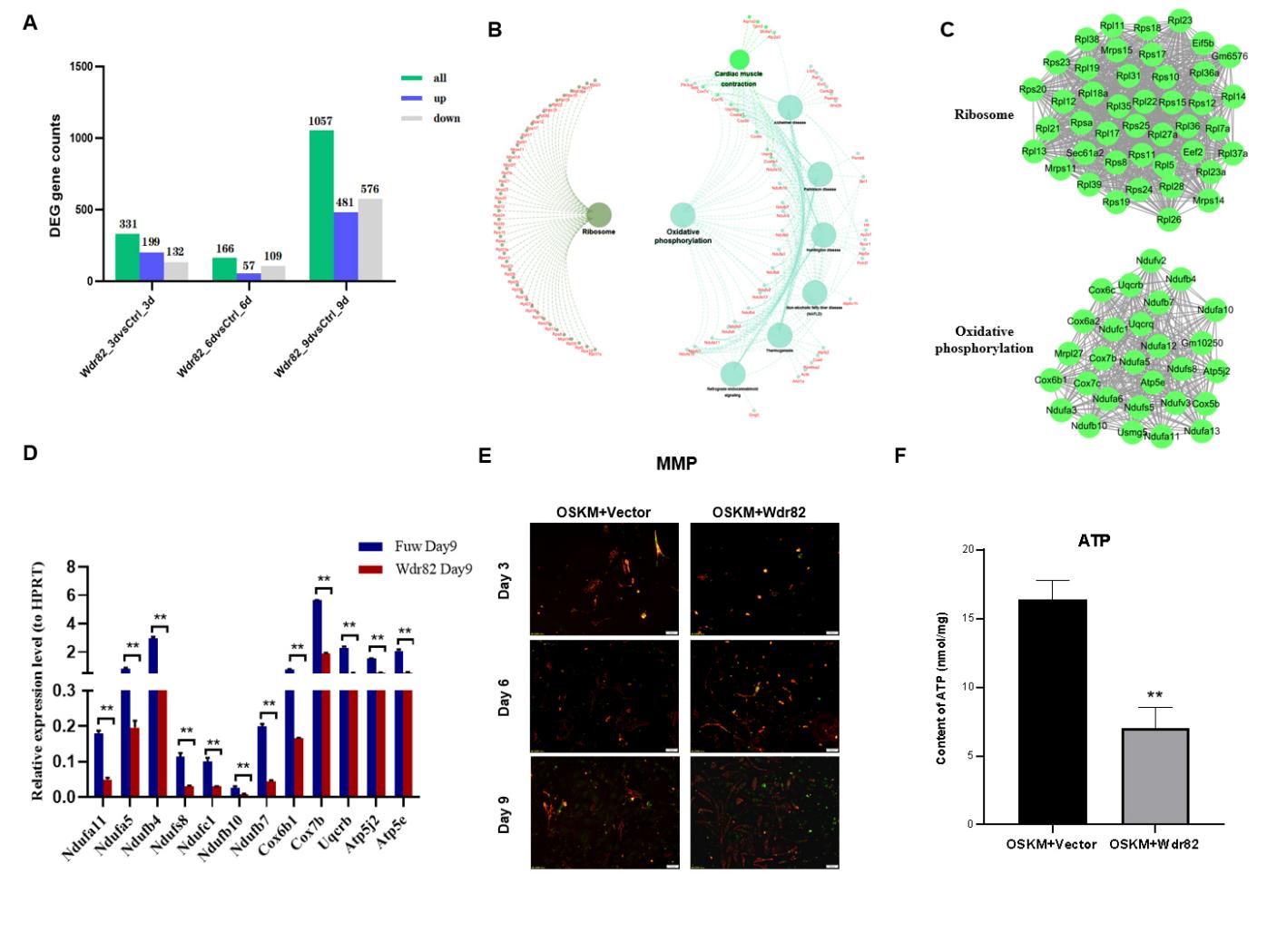


**Figure S6. Transcriptome analysis of OSKM+Wdr82 cells during reprogramming induction.** A. Number of differentially expressed genes were identified by comparing the OSKM+Wdr82 (Wdr82) with its corresponding OSKM control (Ctrl). Samples from reprogramming Day 9 got the most dramatic gene expression difference compared with that of OSKM control. B. The combined analysis of GO and KEGG pathways shows that they are mainly enriched in the biological pathways associated with ribosomes and oxidative phosphorylation. C. The protein-protein interaction network analysis indicates three highly interactive protein clusters at reprogramming Day9, including ribosomes and oxidative phosphorylation. D. RT-PCR results confirmed that the expression of oxidative phosphorylation-related genes upon Wdr82 overexpression (Wdr82) was significantly inhibited during reprogramming induction, compared to its vector control (Fuw). E. JC-1 mitochondrial membrane potential (MMP) assay indicates that cells enter into a lower level metabolic state upon Wdr82+OSKM treatment than OSKM only, during reprogramming, especially at Day 9. F. ATP production were significantly decreased at reprogramming Day 9. * *P* <0.05 and ** *P* <0.01.

**A Reprogramming Day 6 B Reprogramming Day 9**


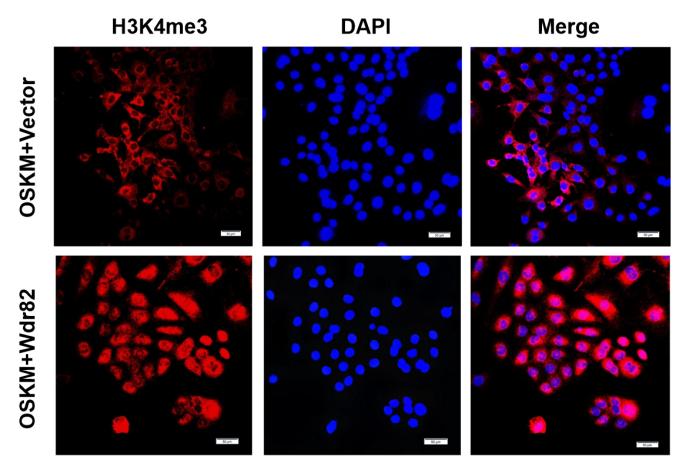

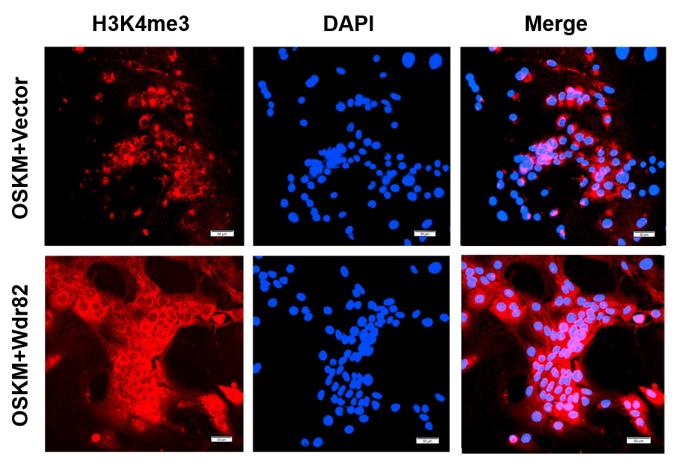


**Figure S7.** The addition of Wdr82 (OSKM+Wdr82) did enhanced the H3K4me3 level in reprogrammed cells at day 6 (A) and day 9 (B), confirming its recruitment role in the Setd1A/B complex, a H3K4me3 methyltransferase complex, during the reprogramming process.

**Table S1.** Sequences of primer pairs used to amplify and contrut the full-length cDNA plasmid specific to indicated genes

| **Construct Name (Gene)** | **Primer sequence (restriction enzyme cutting site)** |
| --- | --- |
| Fuw-TetOn-*Wdr82* | Forward (XbaI): GCTCTAGAATGAAGCTGACCGACAGCG  Reverse (EcoRI): CGGAATTCTCAGTCATCAATGGTGGGC |
| Fuw-TetOn-*Dpy30* | Forward (EcoRI): CCGGAATTCATGGAGTCGGAGCAGATGCT  Reverse (BamHI): CGCGGATCCTCAATTTCGATCTTCAAACTGCG |
| Fuw-TetOn-*Taf7* | Forward (EcoRI): CCGGAATTCATGAGTAAGAACAAAGACGATG  Reverse (BamHI): CGGGATCCTCACTTCTCCAGAAGTGATTC |

**Table S2.** Sequences of DNA oligo used to contrut the plasmid specific *Wdr82* knowdown

| **Construct name** | **Primer sequences** |
| --- | --- |
| sh *Wdr82-*1 | Forward :TTGATCCAGAAGGGTTAATTTTCAAGAGAAATTAACCCTTCTGGATCATTTTTTC  Reverse :TCGAGAAAAAATGATCCAGAAGGGTTAATTTCTCTTGAAAATTAACCCTTCTGGATCAA |
| sh *Wdr82-*2 | Forward :TCAGCCAACACAGTCGTTTATTCAAGAGATAAACGACTGTGTTGGCTGTTTTTTC  Reverse :TCGAGAAAAAACAGCCAACACAGTCGTTTATCTCTTGAATAAACGACTGTGTTGGCTGA |
| sh *Wdr82-*3 | Forward :TACACTCGAAGCTTCATTTATTCAAGAGATAAATGAAGCTTCGAGTGTTTTTTTC  Reverse :TCGAGAAAAAAACACTCGAAGCTTCATTTATCTCTTGAATAAATGAAGCTTCGAGTGTA |
| sh *Wdr82-*4 | Forward:TACGGAGAGAGTGGTATAAATTCAAGAGATTTATACCACTCTCTCCGTTTTTTTC  Reverse:TCGAGAAAAAAACGGAGAGAGTGGTATAAATCTCTTGAATTTATACCACTCTCTCCGTA |

**Table S3.** Primer sequences of OSKM gene expression assessment at reprogramming Day 2

| **Genes** | **Primer sequences** |
| --- | --- |
| Total-*Oct4* | Forward: TCTTTCCACCAGGCCCCCGGCTC  Reverse: TGCGGGCGGACATGGGGAGATCC |
| Total-*Sox2* | Forward: TAGAGCTAGACTCCGGGCGATGA  Reverse: TTGCCTTAAACAAGACCACGAAA |
| Total-*Klf4* | Forward:GCGGGAAGGGAGAAGACAC  Reverse:GGGGAAGACGAGGATGAAGC |
| Total-*c-Myc* | Forward:CAGCGACTCTGAAGAAGAGCA  Reverse:TTGTGCTGGTGAGTGGAGAC |
| qPCR-*Dpy30* | Forward:ACCCTCACTCTGAGTACGGG  Reverse:CCACCTTCTGTTTCGATGACTT |
| qPCR-*Taf7* | Forward:AACAAAGACGATGCGCCTCAT  Reverse:CAGGTTGACATGCCCAGACT |
| qPCR-*Wdr82* | Forward:GACCTCATCAGATACACCCATGC  Reverse:GTCATGCAAGGACAAGTAACGAA |
| *Hprt* | Forward: CACAGGACTAGAACACCTGC  Reverse: GCTGGTGAAAAGGACCTCT |
| *Gapdh* | Forward: TGGCAAAGTGGAGATTGTTGCC  Reverse: AAGATGGTGATGGGCTTCCCG |

**Table S4.** RT-qPCR primer sequences used for expression assessment of pluripotency genes

| **Genes** | **Primer sequences** |
| --- | --- |
| Endogenous-*Oct4* | Forward: TCTTTCCACCAGGCCCCCGGCTC  Reverse: TGCGGGCGGACATGGGGAGATCC |
| Endogenous-*Sox2* | Forward: TAGAGCTAGACTCCGGGCGATGA  Reverse: TTGCCTTAAACAAGACCACGAAA |
| Endogenous-*Klf4* | Forward: GCGAACTCACACAGGCGAGAAACC  Reverse: TCGCTTCCTCTTCCTCCGACACA |
| Endogenous-*c-Myc* | Forward: TGACCTAACTCGAGGAGGAGCTGGAATC  Reverse: AAGTTTGAGGCAGTTAAAATTATGGCTGAAGC |
| *Nanog* | Forward: AGGGTCTGCTACTGAGATGCTCTG  Reverse: CAACCACTGGTTTTTCTGCCACCG |
| *Rex1* | Forward: ACGAGTGGCAGTTTCTTCTTGGGA  Reverse: TATGACTCACTTCCAGGGGGCACT |
| *Utf1* | Forward: GGATGTCCCGGTGACTACGTCTG  Reverse: GGCGGATCTGGTTATCGAAGGGT |

**Table S5.** Primer sequences of marker genes specifically expressed

in differentiated embryonic germ layers

| **Embryonic Germ Layer** | **Marker Genes** | **Primer sequences** |
| --- | --- | --- |
| Ectoderm | *Fgf5* | Forward: AACTCCATGCAAGTGCCAAAT  Reverse: CGGACGCATAGGTATTATAGCTG |
|  | *Kdr* | Forward: TTTGGCAAATACAACCCTTCAGA  Reverse: GCAGAAGATACTGTCACCACC |
| Mesoderm | *Nkx2.5* | Forward: GACAAAGCCGAGACGGATGG  Reverse: CTGTCGCTTGCACTTGTAGC |
|  | *Myf5* | Forward: AAGGCTCCTGTATCCCCTCAC  Reverse: TGACCTTCTTCAGGCGTCTAC |
|  | *MyoD* | Forward: CCACTCCGGGACATAGACTTG  Reverse: AAAAGCGCAGGTCTGGTGAG |
| Endoderm | *Sox17* | Forward: GATGCGGGATACGCCAGTG  Reverse: CCACCTCGCCTTTCACCTTTA |
|  | *Gata4* | Forward: CCCTACCCAGCCTACATGG  Reverse: ACATATCGAGATTGGGGTGTCT |
|  | *Sox7* | Forward: CAAGATGCTGGGTAAGTGCG  Reverse: CCTTTCCGAACAGCGTTGTC |
| Trophoblast | *Eomes* | Forward: GAGCTTCAACATAAACGGACTCAA  Reverse: CGGCCAGAACCACTTCCA |
|  | *Cdx2* | Forward: CAAGGACGTGAGCATGTATCC  Reverse: GTAACCACCGTAGTCCGGGTA |

**Table S6.** Sequences of primer pairs used to confirmation the expression difference of OXPHOS genes during reprogramming process

| **Gene Name** | **Primer sequences** |
| --- | --- |
| *Ndufa11* | Forward: GGTGAAGCGGTTCTTTGAATCT  Reverse: GAGCCGATTATGCCGCAAATG |
| *Ndufa5* | Forward: ATGGCGGGCTTGCTGAAAA  Reverse: GCTGCATGTTTAGGAAAGTGCTT |
| *Ndufb4* | Forward: CTTGATTCGCTGGACCTATGC  Reverse: GGAGTGGGCCTGAAATTAGGA |
| *Ndufs8* | Forward: AGTGGCGGCAACGTACAAG  Reverse: TCGAAAGAGGTAACTTAGGGTCA |
| *Ndufc1* | Forward: GTAGTGCTGCGCTCGTTTTC  Reverse: CCAACCAGTTAGGTTTGGCAT |
| *Ndufb10* | Forward: GATTCTTGGGACAAGGATGTGT  Reverse: CCTTCGTCAAGTAGGTGATGGG |
| *Ndufb7* | Forward: CGGCGCTATCTGTGGGATG  Reverse: CTGTCTCGCTTGCACTTCAG |
| *Cox6b1* | Forward: ACTACCTGGACTTCCACCG  Reverse: ACCCATGACACGGGACAGA |
| *Cox7b* | Forward: TTGCCCTTAGCCAAAAACGC  Reverse: TCATGGAAACTAGGTGCCCTC |
| *Uqcrb* | Forward: GGCCGATCTGCTGTTTCAG  Reverse: CATCTCGCATTAACCCCAGTT |
| *Atp5j2* | Forward: TGCCGAGCTGGATAATGATGC  Reverse: ACCATGCTAATCCCCGAGATG |
| *Atp5e* | Forward: CAGGCTGGACTCAGCTACATC  Reverse: GTTCGCTTTGAACTCGGTCTT |
| *Hk2* | Forward: TGATCGCCTGCTTATTCACGG  Reverse: AACCGCCTAGAAATCTCCAGA |
| *Pk2* | Forward: GCCGCCTGGACATTGACTC  Reverse: CCATGAGAGAAATTCAGCCGAG |
| *Pfk* | Forward: TGTGGTCCGAGTTGGTATCTT  Reverse: GCACTTCCAATCACTGTGCC |
| *Cpt1α* | Forward: CTCCGCCTGAGCCATGAAG  Reverse: CACCAGTGATGATGCCATTCT |
| *Cpt2* | Forward: CAGCACAGCATCGTACCCA  Reverse: TCCCAATGCCGTTCTCAAAAT |
| *Ldhα* | Forward: TGTCTCCAGCAAAGACTACTGT  Reverse: GACTGTACTTGACAATGTTGGGA |
